# Low CD8+ Density Variation and R1 Surgical Margin as Independent Predictors of Early Post-Resection Recurrence in HCC Patients Meeting Milan Criteria

**DOI:** 10.3390/curroncol31090394

**Published:** 2024-09-10

**Authors:** Rokas Stulpinas, Ieva Jakiunaite, Agne Sidabraite, Allan Rasmusson, Dovile Zilenaite-Petrulaitiene, Kestutis Strupas, Arvydas Laurinavicius, Aiste Gulla

**Affiliations:** 1 Institute of Biomedical Sciences, Department of Pathology and Forensic Medicine, Faculty of Medicine, Vilnius University, 03101 Vilnius, Lithuania; 2 National Center of Pathology, Affiliate of Vilnius University Hospital Santaros Clinics, 08406 Vilnius, Lithuania; 3 Faculty of Medicine, Vilnius University, 03101 Vilnius, Lithuania; 4 Institute of Informatics, Faculty of Mathematics and Informatics, Vilnius University, 03225 Vilnius, Lithuania; 5 Institute of Clinical Medicine, Centre for Visceral Medicine and Translational Research, Faculty of Medicine, Vilnius University, 01513 Vilnius, Lithuania; 6 Department of Surgery, School of Medicine and Health Sciences, The George Washington University, Washington, DC 20052, USA

**Keywords:** CD8, digital pathology, hepatocellular carcinoma (HCC), tumor-infiltrating lymphocytes, Milan criteria, liver transplantation

## Abstract

Our study included 41 patients fulfilling the Milan criteria preoperatively and aimed to identify individuals at high risk of post-resection HCC relapse, which occurred in 18 out of 41 patients (43.9%), retrospectively. We analyzed whole slide images of CD8 immunohistochemistry with automated segmentation of tissue classes and detection of CD8+ lymphocytes. The image analysis outputs were subsampled using a hexagonal grid-based method to assess spatial distribution of CD8+ lymphocytes with regards to the epithelial edges. The CD8+ lymphocyte density indicators, along with clinical, radiological, post-surgical and pathological variables, were tested to predict HCC relapse. Low standard deviation of CD8+ density along the tumor edge and R1 resection emerged as independent predictors of shorter recurrence-free survival (RFS). In particular, patients presenting with both adverse predictors exhibited 100% risk of relapse within 200 days. Our results highlight the potential utility of integrating CD8+ density variability and surgical margin to identify a high relapse-risk group among Milan criteria-fulfilling HCC patients. Validation in cohorts with core biopsy could provide CD8+ distribution data preoperatively and guide preoperative decisions, potentially prioritizing liver transplantation for patients at risk of incomplete resection (R1) and thereby improving overall treatment outcomes significantly.

## 1. Introduction

Liver cancer is one of the five most lethal malignancies and ranks sixth in global morbidity rates [1,2]. The most common type of liver cancer is hepatocellular carcinoma (HCC), comprising over 90% of cases, with only 10–15% of patients achieving a five-year survival rate worldwide [3]. The incidence of HCC increases annually; furthermore, relapse occurs in 30–50% of patients during the first two years after resection [1,4]. Known risk factors for early relapse include male gender, large tumor size, tumor multifocality, high serum alpha-fetoprotein level and many others [4,5].

The complex tumor microenvironment plays a critical role in the progression and metastasis of HCC [6]. The host immune response, particularly involving CD8+ lymphocytes or cytotoxic T cells, can significantly influence tumor recurrence and patient survival outcomes in HCC [7,8]. Higher densities of CD8+ cells often indicate a robust anti-tumoral immune response that can suppress tumor growth and prevent recurrence. While some earlier studies found no prognostic value in CD8+ cell densities [9], others suggested that high tumor infiltrating lymphocyte (TIL) levels could contribute to HCC development and relapse [10]. However, more recent evidence indicates a positive association between high TIL densities and improved outcomes [8,11]. Emerging research not only examines the average density of tumor-infiltrating lymphocytes but also the spatial heterogeneity of their infiltration. For example, the study by Li et al. determined that both intratumoral and peritumoral lymphoid clusters (tertiary lymphoid structures (TLS)) play crucial roles in HCC, with high peritumoral TLS density correlating with increased immune cell infiltration and a better patient prognosis [12]. Understanding and measuring the precise spatial distribution of tumor-infiltrating lymphocytes is essential for advancing treatment strategies and improving outcomes for HCC patients.

Currently, liver transplantation (LT) is regarded as the first-line treatment option for patients with HCC who meet the Milan criteria [13]. Liver transplantation, when performed as an initial treatment, has the potential to simultaneously remove both the tumor and the underlying disease, typically cirrhosis, thereby offering superior long-term outcomes compared to liver resection (LR). This advantage is demonstrated by a 5-year disease-free survival (DFS) rate of up to 96.8% for LT, compared to 64.3% for LR, and a nearly 50% reduction in mortality rate, although some of the numbers do seem overly optimistic and could be biased [14,15]. Even though the risk of HCC recurrence after LR is threefold that of LT, a shortage of cadaveric organs limits the selection of this therapeutic modality [15]. Salvage liver transplantation (SLT) serves as a crucial alternative curative approach, demonstrating comparable DFS rates between primary liver transplantation (PLT) and SLT–LR groups [16].

Since the introduction of the Milan criteria in 1996 (a single tumor with a diameter ≤5 cm; or no more than three tumors, each ≤3 cm in size; and no vascular invasion; and no extrahepatic involvement) into clinical use, survival rates after LT for HCC have improved significantly [17]. Despite the strict adherence to these criteria, tumor recurrence occurs in up to 20% of HCC patients who have undergone LT, with 75% of the recurrences emerging during the first 2 years after the LT [18,19]. Stratifying individuals who meet the Milan criteria into distinct risk categories may aid the decision-making process for LT as the next line of therapeutic intervention.

This study aims to improve the prediction of post-resection HCC recurrence in patients meeting the Milan criteria preoperatively using paraffin-embedded tissue, CD8 immunohistochemistry, AI tissue segmentation and hexagonal grid subsampling-based image analytics. If further validated on biopsy material, our findings suggest that integrating CD8+ T cell density variability into preoperative predictive models could aid in decision-making, particularly in considering liver transplantation for patients at risk of incomplete (R1) resection.

## 2. Materials and Methods

### 2.1. Study Population

The cohort for this retrospective study comprised consecutive patients, totaling 41 individuals, who underwent liver resection for HCC between 2007–2020 at Vilnius University Hospital Santaros Clinics (Vilnius, Lithuania) and, at the time of surgery, fulfilled the Milan criteria for liver transplantation preoperatively. The resected tissue was processed, analyzed and archived in the National Center of Pathology (Vilnius, Lithuania). The study was approved by the Vilnius Regional Biomedical Research Ethics Committee (permit number 2021/6-1354-843). 

### 2.2. Immunohistochemistry

A pathologist (RS) reviewed the archived slides stained with hematoxylin and eosin to identify the optimal formalin-fixed paraffin-embedded (FFPE) tissue block. The selected samples, cut to 3 µm in thickness, were mounted on positively charged slides and immunohistochemically stained for CD8 using Dako’s C8/144B antibody (dilution 1:100, Dako, Glostrup, Denmark). Staining was performed on a Roche Ventana BenchMark ULTRA automated stainer with the ultraView Universal DAB Detection kit (Ventana Medical Systems, Oro Valley, AZ, USA).

### 2.3. Digital Image Analysis and Indicator Extraction

The detailed process workflow is explained in our previously published paper [20]. Briefly, slides were digitized at 20× magnification (0.5 µm per pixel) using an Aperio^®^ AT2 DX scanner (Leica Aperio Technologies, Vista, CA, USA). A pathologist (RS) marked the tumor and residual liver parenchyma areas by placing annotations. The HALO^®^AI (Indica Labs, Albuquerque, NM, USA) system was subsequently trained to segment tissue into epithelial/hepatocytes, stroma and background/debris classes, with CD8+ cell segmentation performed using the HALO^®^ Multiplex IHC algorithm. We further processed the HALO^®^AI outputs by using a hexagonal grid tiling method (having a side length of 65 µm in this study) as described by Rasmusson et al. [21].

**Figure 1:**
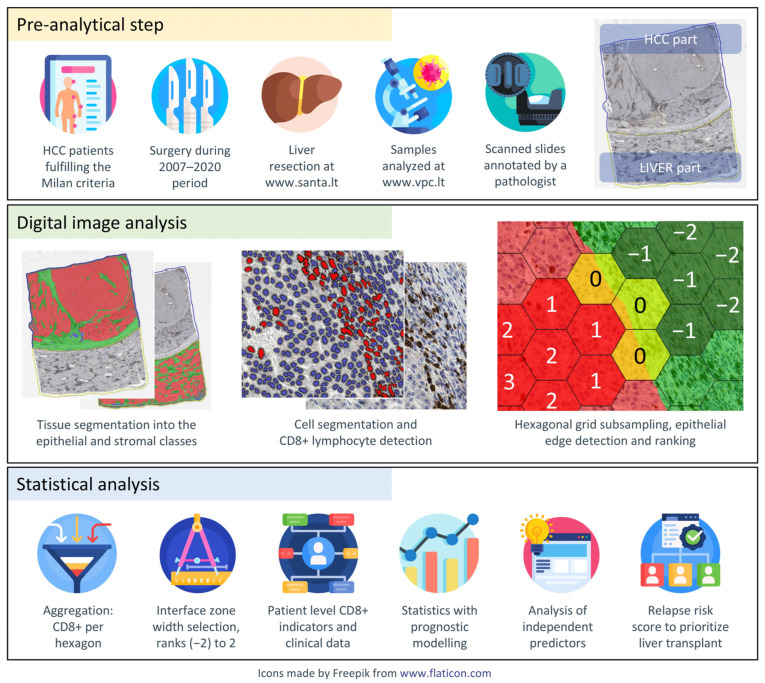
Study workflow. Pre-analytical step: HCC patients meeting the Milan criteria underwent liver resection between 2007–2020. Samples were analyzed and scanned slides were annotated by a pathologist. Digital image analysis: tissue samples were segmented into epithelial and stromal classes, and CD8+ lymphocytes were detected using HALO^®^AI (Indica Labs, USA). Hexagonal grid subsampling was applied for epithelial edge detection. Statistical analysis: aggregated CD8+ data per hexagon were combined with clinical data for prognostic modeling. Independent predictors were analyzed, and a relapse risk score was developed to prioritize liver transplant candidates.

The number of CD8+ cells and the area of tissue classes in each hexagon were aggregated for the malignant (HCC) and non-malignant (residual liver parenchyma) parts of the slide. Based on the abrupt change in tissue class proportions across the grid, we identified hexagons on the extracted epithelial edge and assigned them a rank of 0. The remaining epithelial hexagons (representing HCC or liver depending on the area analyzed) are assigned positive ranks, whereas hexagons on the stromal side received negative ranks corresponding to their distance from the nearest edge. Immune response indicators were extracted from five hexagon-wide interface zones (ranks −2, −1, 0, 1, 2) of the non-neoplastic liver and HCC to reflect CD8+ cell density profiles in both tissue compartments, including mean density, standard deviation (SD), the center of mass and immunodrop ratio (the ratio of CD8+ cell quantities at ranks −1 and 1, indicating abrupt change in cell density at the tumor edge) (see Figure 1).

### 2.4. Statistical Analysis and Modeling

To meet the assumptions of normality and homoscedasticity, the initial aggregated data underwent a logarithmic transformation of the CD8+ density values. For improved readability, the ‘log’ prefix is omitted in the subsequent text. Significance levels were set at *p* < 0.05. Univariate Cox regression was used to evaluate the prognostic significance of conventional clinicopathologic predictors, which were represented by either continuous or categorical variables. Then, a multivariate Cox regression with stepwise likelihood ratio (LR) testing was performed to assess the independent prognostic value of the CD8+ lymphocyte distribution indicators, represented as continuous variables, in the context of the statistically significant conventional predictors identified in the univariate analysis. An integrated Recurrence Risk Score was derived by summing the negative impacts of the independent predictors of recurrence-free survival (RFS). RFS was estimated using the Kaplan–Meier method, followed by log-rank testing to compare the statistical significance of the RFS distributions. SAS (version 9.4; SAS Institute Inc., Cary, NC, USA) and R version 4.3.2 (R Foundation for Statistical Computing, Vienna, Austria), along with the survminer and ggplot2 packages, were employed for data analysis in this study.

## 3. Results

### 3.1. Summary of Patient Cohort Characteristics

Patient gender, age, tumor grade, pT stage, intravascular invasion (as reported in the pathology report), resection margin, number of tumors, largest tumor size, the presence of cirrhosis, duration of surgery, hospitalization time and the date of HCC recurrence were collected from hospital records and are presented in Table 1.

### 3.2. Predictors of Recurrence-Free Survival

The resulting Cox proportional hazard model of the Milan criteria-fulfilling patient cohort consisted of two independent predictors of a shorter RFS after HCC resection (see Table 2). One predictor was a histologic feature of immune response, specifically a low SD of CD8+ density along the tumor edge (HR = 0.246 (95% CI 0.078–0.779), *p* = 0.0171, see Figure S1 “Representative images demonstrating CD8+ cell density variation at the tumor edge”), the second—a conventional parameter, the R1 resection as defined in the final pathology report (HR = 7.162 (95% CI 2.213–23.185), *p =* 0.0010). None of the other patient or tumor characteristics had a statistically significant impact on RFS.

**Figure 2:**
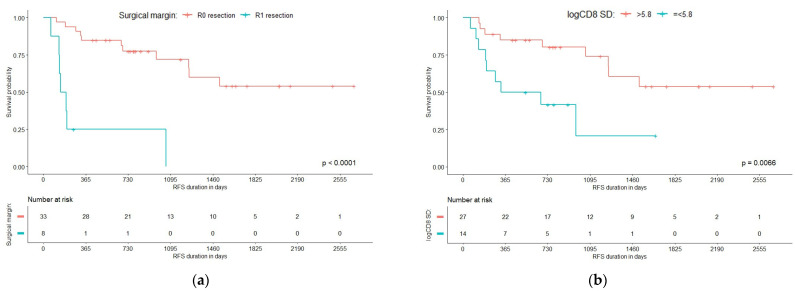
The R1 resection (**a**) and low standard deviation of CD8+ density along the tumor edge (**b**) as univariate predictors for shorter RFS.

Panel (a) of Figure 2 displays the survival probabilities over time for patients categorized by their resection status: R0 (complete resection) and R1 (incomplete resection). The *p*-value for the comparison between R0 and R1 groups is very low (*p* < 0.0001), indicating that patients with complete resection (R0) have a longer RFS compared to those with incomplete resection (R1). Panel (b) of Figure 2 illustrates the RFS probabilities for patients based on the SD of CD8 density at the tumor edge, with groups divided into low (<5.8) = 1 and high (>5.8) = 0. The *p*-value for this comparison is 0.0066, indicating a statistically significant difference between the two groups and implying that a low SD of CD8+ density along the tumor edge has a negative impact on RFS.

### 3.3. Recurrence Risk Score

**Figure 3:**
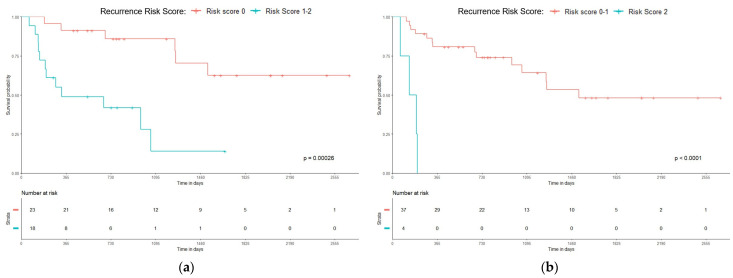
HCC Recurrence Risk Score: (**a**) 0 vs. 1–2 (*p* = 0.00026) and (**b**) 0–1 vs. 2 (*p* < 0.0001).

Based on the findings, a combined prognostic Relapse Score was constructed by summing up the contributions from both independent variables and assigning a value of 1 for a poor and 0 for a good prognosis. Since there are two independent predictors with either a 0 or 1 value, the possible score for each patient is 0 (0 + 0), 1 (1 + 0 or 0 + 1) or 2 (1 + 1). The Kaplan–Meier survival curves for these groups with significant RFS differences are shown in Figure 3. The chart suggests that individuals who exhibit both adverse predictors experience a 100% relapse risk within a relatively short timeframe—less than 200 days. 

## 4. Discussion

Our study explored the link between the spatial distribution of CD8+ cytotoxic T cells, the established clinicopathologic adverse factors and the risk of recurrence following liver resection for HCC. The finding that a higher variance (SD) of CD8+ T lymphocyte density at the tumor edge is associated with longer RFS in HCC patients is intriguing. While generally denser and more uniform infiltration of CD8+ T cells is linked to better cancer patient outcomes [22,23,24], our data suggest a more nuanced relationship. Some studies in other cancers have shown similar trends: for example, Krijgsman et al. (also utilizing a deep learning tissue classifier combined with immunohistochemistry) discovered that breast cancer patients with high variation (SD) of CD8+ cell density had longer overall survival (OS) [25]. A potential explanation is that the high SD of CD8+ lymphocyte density mathematically reflects the presence of localized, denser immune cell clusters. These clusters, in some cases, might represent the well-established tertiary lymphoid structures (TLS) known to be favorable prognostic markers in HCC [16,26,27,28]. Uniformly distributed CD8+ lymphocytes at the tumor edge could on the other hand be exhausted T cells that lack the ability to effectively induce an anti-tumoral response [29]. Furthermore, chronic HBV and HCV infection that often precede HCC can lead to the production of immunosuppressive cytokines within the liver microenvironment, which impairs the function of T cells, further contributing to exhaustion [30,31,32]. To validate this hypothesis, future studies could analyze the expression of immune checkpoint molecules on these CD8+ T cells.

Liver transplantation is the preferred treatment for selected HCC patients, offering markedly improved outcomes in 5-year OS and RFS rates (64.83% and of 70.20%, respectively), compared to liver resection (OS: 50.83%; RFS: 34.46%) [33]. Meanwhile, as patients remain on the waiting list for a donor, interim management strategies such as liver resection, ablation and transarterial interventions are employed to control disease progression [34]. Liver resection is considered a curative procedure for individuals diagnosed with HCC, though it is associated with significant recurrence rates, with 60% of cases relapsing within three years. Moreover, resection yields tissue essential for the pathological evaluation of key independent predictors of HCC recurrence and post-transplantation survival, namely the presence of satellite nodules, the degree of tumor differentiation and microvascular invasion [35]. Although the ideal margin width remains a topic of debate, surgical resection for HCC is generally not advised for patients whose tumors cannot be completely removed with negative (R0) margins [35].

The identification of a positive margin (R1) as a significant independent predictor of a shorter RFS is consistent with the established knowledge that incomplete removal of neoplastic tissue increases the likelihood of recurrence. In our cohort, when a safe surgical margin was unattainable based on intraoperative findings, surgical resection was carried out, ensuring the complete macroscopic removal of the tumor. The overall recurrence rate was observed to be 43.9%, with relapses occurring significantly more frequently in the R1 group (87.5%) compared to the R0 group (33.3%), *p* = 0.0133. Numerous studies have evaluated the influence of surgical margins on the outcomes of HCC following liver resection [36,37,38]. Although these studies vary in nature, they consistently report that the width of the resection margin does not impact postoperative recurrence rates following hepatectomy for HCC. Poon et al. concluded that a positive margin correlates with an increased risk of postoperative recurrence and is frequently associated with underlying venous invasion or the presence of microsatellites [39].

The Recurrence Risk Score, combining CD8+ density variability (represented by the standard deviation) at the tumor edge and resection margin status, offers a potential tool to identify patients at high risk of early relapse. In addition, although formally eligible for transplantation, patients over 70 years of age demonstrate poorer post-transplantation outcomes than younger cohorts, warranting cautious consideration of liver transplantation in this age group [40]. Liver transplantation should always be considered for eligible patients; however, investigating the factors influencing CD8+ cell distribution and their utility as prognostic biomarkers for recurrence and long-term survival is crucial prior to relying on them for treatment decisions.

### Limitations

This study is subject to several limitations that should be acknowledged. First, the sample size of 41 patients, particularly within certain subgroups (*n* = 4), is relatively small, which may limit the generalizability of our findings. Additionally, the still somewhat heterogeneous nature of the study cohort could introduce variability that impacts the robustness of the results. These factors necessitate caution when interpreting the data. Future validating studies with larger cohorts and/or biopsy core tissues are highly encouraged.

## 5. Conclusions

In conclusion, our study contributes to the understanding of the immune response’s role in predicting HCC recurrence following liver resection. By integrating CD8+ spatial distribution indicators with resection margin status, we present a potentially novel methodology for identifying high-risk HCC patients who meet the Milan criteria preoperatively. Subsequent validation in cohorts using core biopsy material could provide preoperative CD8+ distribution data, thereby guiding preoperative decisions and potentially prioritizing liver transplantation for patients at risk of incomplete resection (R1). This approach could significantly improve overall treatment outcomes. Although significant trends were observed, further research with larger and more diverse cohorts is necessary.

## Figures and Tables

**Table 1:** Patient demographics and clinical characteristics.

Characteristic	Value (%)
Patients	41 (100%)
Age, years: Mean (SD) Median (IQR)	64.4 (9.16) 66 (12)
Age distribution <50 years 50–59 years 60–69 years 70–79 years ≥80 years	1 (2.4%) 10 (24.4%) 18 (43.9%) 11 (26.8%) 1 (2.4%)
Gender Males Females	28 (68.3%) 13 (31.7%)
Tumor grade G1 G2 G3	4 (9.8%) 29 (70.7%) 8 (19.5%)
pT stage: pT1 pT2	27 (65.9%) 14 (34.1%)
Intravascular invasion LVI present LVI absent	12 (29.3%) 29 (70.7%)
Resection margin R0 R1	33 (80.5%) 8 (19.5%)
Number of tumors One tumor Two tumors Three tumors	32 (78.0%) 7 (17.1%) 2 (4.9%)
Tumor size in the pathology report, mm Mean (SD) Median (IQR)	28 (11) 25 (20)
Recurrences HCC recurrence No recurrence	18 (43.9%) 23 (56.1%)
RFS time, days Mean (SD) Median (IQR)	904.4 (702.9) 749 (933)

**Table 2:** Independent predictors of recurrence-free survival (RFS) in Milan criteria-fulfilling patients based on multivariate Cox regression modelling.

Indicator	DF	Parameter Estimates	Standard Error	Chi-Square	*p*-Value	Hazard Ratio	95% Hazard Ratio Confidence Limits	
R1 resection	1	1.96882	0.59935	10.7909	0.0010	7.162	2.213	23.185
SD of CD8 at tumor edge	1	−1.40113	0.58769	5.6842	0.0171	0.246	0.078	0.779

Likelihood Ratio Test: Chi-square 17.7246, *p* = 0.0001.

## Data Availability

The data presented in this study are available on request from the corresponding author. The data are not publicly available due to permit restrictions.

## Supplementary Materials

The following supporting information can be downloaded at: https://www.mdpi.com/article/10.3390/curroncol31090394/s1, Figure S1: Representative images demonstrating CD8+ cell density variation at the tumor edge.
