# Supplementary figures and images for: Low CD8+ Density Variation and R1 Surgical Margin as Independent Predictors of Early Post-Resection Recurrence in HCC Patients Meeting Milan Criteria
Source: Curr Oncol. 2024 Sep 10;31(9):5344–53. doi: 10.3390/curroncol31090394 (PMC11431076; doi:10.3390/curroncol31090394)

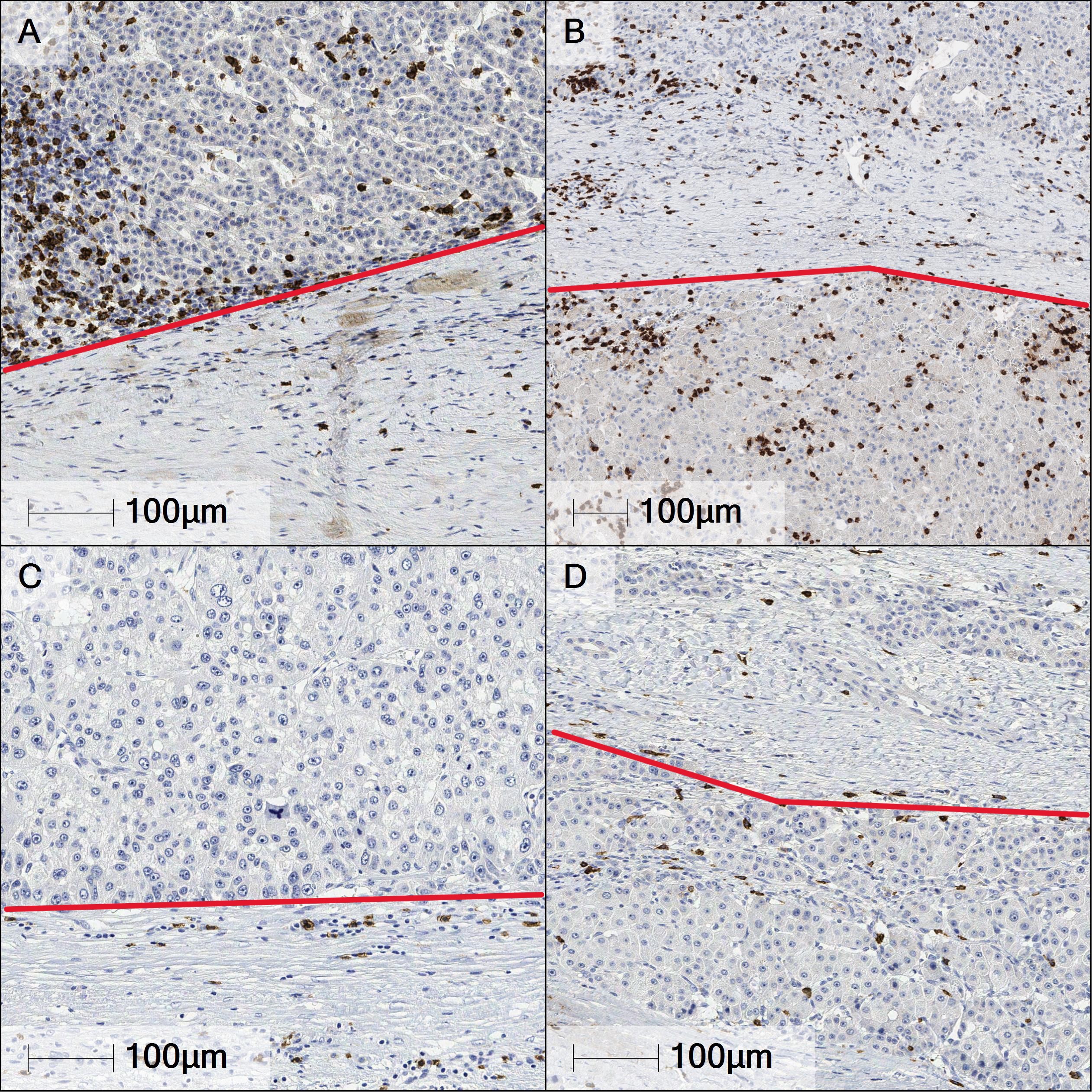

Supplement: Supplementary file 1 [file curroncol-31-00394-s001.zip › curroncol-3139124-supplementary.jpg]
